# Primary ventral and incisional hernias: comprehensive review

**DOI:** 10.1093/bjsopen/zrae145

**Published:** 2025-02-03

**Authors:** Nadia A Henriksen, Heather Bougard, Mário R Gonçalves, William Hope, Ritu Khare, Jenny Shao, Andrea C Quiroga-Centeno, Eva B Deerenberg

**Affiliations:** Department of Gastrointestinal and Hepatic Diseases, Herlev Hospital, University of Copenhagen, Herlev, Denmark; Department of Surgery, New Somerset Hospital, University of Cape Town, Cape Town, South Africa; Department of Surgery, Hospital de Braga, Braga, Portugal; Department of Surgery, Novant Health New Hanover Regional Medical Center, Wilmington, North Carolina, USA; Department of Surgery, Kings College Hospital, Dubai, United Arab Emirates; Department of Surgery, University of Michigan, Ann Arbor, Michigan, USA; Department of Surgery, Universidad Industrial de Santander, Bucaramanga, Colombia; Department of Surgery, Franciscus en Vlietland, Rotterdam, The Netherlands

## Abstract

**Background:**

Primary ventral and incisional hernias are frequent conditions that impact the quality of life of patients. Surgical techniques for ventral hernia repair are constantly evolving and abdominal wall surgery has turned into a highly specialized field.

**Methods:**

This is a narrative review of the most recent and relevant literature on the treatment of primary ventral and incisional hernias performed by eight experts in ventral hernia surgery from across the world and includes review of classification systems, preoperative measures, descriptions of surgical techniques, and postoperative complications.

**Results:**

Repairs of primary ventral and incisional hernias range from simple open procedures in healthy patients with small defects to complex procedures when patients are co-morbid and have large defects. Optimizing patient-related risk factors before surgery is important to decrease complication rates. Surgical repair techniques from open repairs to minimally invasive procedures are described in detail in the review. Minimally invasive techniques are technically more demanding and take longer, but decrease the risk of surgical-site infections and shorten the duration of hospital stay.

**Conclusion:**

Treatment of ventral hernias aims to improve the quality of life of patients. The risks and benefits of procedures should be weighed against patients’ complaints and co-morbidities. Optimizing patient-related risk factors before surgery is important.

## Teaser

This comprehensive review gives an overview of the classification, diagnosis, preoperative considerations, treatment strategies, and the most used surgical procedures for primary ventral and incisional hernias.

## Introduction

Ventral hernias, including primary and incisional hernias, are common disease pathologies in surgical practice. A primary hernia is a spontaneous defect in the abdominal wall, whereas an incisional hernia is a hernia that occurs at the site of a previous incision and includes recurrent hernias. An incisional hernia is one of the most frequent complications of abdominal surgery and it is estimated that up to 30% of patients will develop a symptomatic hernia 2 years after open abdominal surgery^1^.

Ventral hernias can have a significant impact on the quality of life of patients due to bulging, discomfort, pain, and cosmetic concerns^2^. Moreover, significant morbidity such as bowel incarceration and bowel ischaemia may be a potential complication caused by ventral hernias. Repair of primary ventral and incisional hernias can be complex, with complication rates ranging from 4–48% and recurrence rates of up to 50%, depending on follow-up time^3^. These rates of complication and recurrence can further increase after each subsequent failed repair^4^. Lastly, ventral hernia repairs are associated with a significant cost for healthcare systems^5^. These costs can be potentiated by patient-related risk factors such as smoking, obesity, and diabetes, which have been shown to increase complication and recurrence rates, therefore adding to the cost and complexity of the procedure^6^. Research by the French Surgical Society concluded that prevention and decreasing the incidence of just 5% of incisional hernias per year would result in a decrease in healthcare costs of 4 million euros per year^7^.

Patient selection, preoperative optimization, surgeon specialization, and choice of the optimal surgical approach are key factors to improve outcomes and reduce overall societal costs. Surgical techniques for ventral hernia repair are constantly evolving and abdominal wall surgery has turned into a highly specialized field^8^. The aim of this comprehensive review is to give an overview of the classification, diagnosis, preoperative considerations, treatment strategies, and the most used surgical procedures for primary ventral and incisional hernias. This was performed as a narrative review of the current literature and concepts around ventral and incisional hernia management by eight surgeons with a special interest and research experience in hernia management from Northern and Southern Europe, North and South America, Asia, and Africa.

## Definitions and classification systems

The purpose of classification systems is to have a unified terminology that is widely accepted, helps in the reporting and recording of data, and standardizes comparison across different studies and their results. As the description and classification of hernias aim to determine optimal management, several variables, including hernia and patient characteristics, need to be considered. However, including many variables in the classification of abdominal wall hernias could make the classification system more complicated and difficult to standardize. As various classifications have been proposed, a description of the most widely used definitions and classification systems for primary and incisional hernias is provided below.

### Classification of primary ventral hernias

The European Hernia Society (EHS) classification provides a simple, practical, and reproducible method to classify primary abdominal wall and incisional hernias^9^. A primary hernia is defined as spontaneous herniation of intra-abdominal contents through a defect in the abdominal wall that is not related to a scar from surgery or trauma. The EHS recommends location and size as the only two variables to classify primary abdominal wall hernias. Based on the location, there are two midline hernias (epigastric and umbilical) and two lateral hernias (Spigelian and lumbar).

Primary hernias are usually round or oval, and their size is defined as the maximum transverse diameter or width of the hernia. The EHS and Americas Hernia Society (AHS) guidelines for umbilical and epigastric hernia repair proposed a size classification based on the suggested treatment strategies: less than 1 cm, small; 1–4 cm, medium; and greater than 4 cm, large^10^. For lateral primary hernias, typically, the defect size classification from the EHS classification system is utilized: less than 2 cm, small; 2–4 cm, medium; and greater than 4 cm, large^9^.

### Classification of incisional hernias

An incisional hernia is defined as any abdominal wall gap with or without a bulge in a postoperative scar perceptible or palpable on clinical examination or imaging^11^. Primary hernias that have undergone one or more surgeries previously are also classified as incisional hernias. As per EHS consensus, the location and size of the incisional hernia defect are essential for classification^9^.

**Fig. 1:**
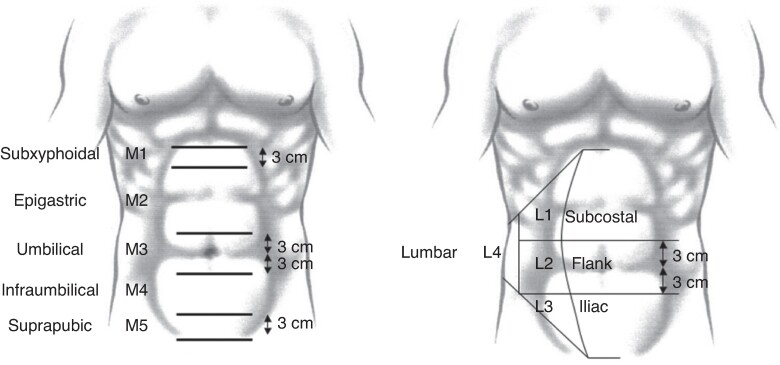
European Hernia Society classification of the location of incisional hernias on the abdominal wall Reproduced from Muysoms *et al*.^134^.

To define the location of an incisional hernia, the abdomen is divided into a medial zone and a lateral zone (*Fig. 1*). Incisional hernias located in the midline or medial zones are coded as ‘M’, whereas those in the lateral zone are coded as ‘L’. The cranial limit of the medial zone is the xiphoid, the caudal limit is the pubis, and the lateral limits are the abdominal rectus sheath. Based on these borders, the medial zone is divided into five zones (M1 to M5). When a hernia is present in two or more zones, all the involved zones should be mentioned. Incisional hernias caused by two or more different incisions should be classified separately.

Regarding the lateral zone, the cranial limit is the costal margin, the caudal limit is the inguinal region, the medial limit is the lateral border of the rectus sheath, and the lateral limit is the lumbar region. It is divided into four zones (L1 to L4).

**Fig. 2:**
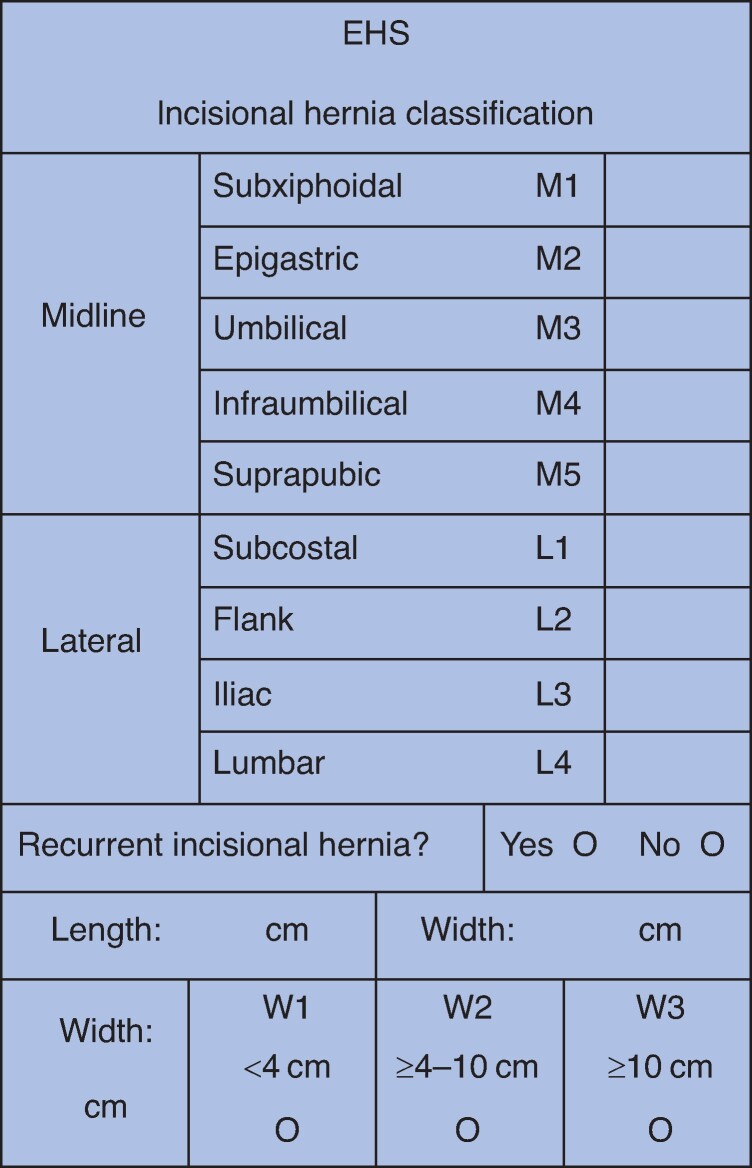
European Hernia Society schematic classification of incisional hernias on the abdominal wall Reproduced from Muysoms *et al*.^134^.

The shape of incisional hernias is usually quite variable and, therefore, size estimation is not straightforward. Both the width and length of the hernial fascial defect should be measured. For classification, the widest transverse diameter of the hernial defect should be used (W1, less than 4 cm; W2, 4–10 cm; and W3, greater than 10 cm) (*Fig. 2*). Multiple defects originating from one incision should be classified as one hernia.

While a history of past repair is an important variable, to simplify the system, this has not been included in the EHS classification. On the other hand, although parastomal hernias are also considered a type of incisional hernia, they are excluded from the EHS classification as they are a distinct type of hernia with their own specific and different surgical techniques. Parastomal hernias are outside the scope of this review and are not addressed in this paper.

### Classification systems for predicting complications after surgery

There are several different classification systems for predicting potential complications after hernia surgery and the following systems are detailed here as they seem to be the ones that are most often referred to: the Ventral Hernia Working Group classification, the Hernia-Patient-Wound (HPW) classification, and the Carolinas equation for Determining Associated Risks (CeDAR). All these classification systems serve as a tool for the surgeon to assess potential modifiable risks for patients seeking surgical repair and are easy to use. Outside of these variables, other predictors for potential complications relate to hernia complexity and loss of domain, which are described below.

The Ventral Hernia Working Group classification was first described in 2010, and modified in 2013, and is a grading system for ventral hernias for stratifying patients at risk of wound complications and recurrences^12^. Patients with hernias are divided into three groups: ‘grade 1—low risk’, for healthy patients with no history of wound infection; ‘grade 2—co-morbid’, for patients with diabetes, chronic obstructive pulmonary disease, immunosuppression, smoking, or obesity; and ‘grade 3—contaminated’, for patients with wound contamination^13^. Grade 3 is further subdivided into A, B, and C according to level of contamination (*Table 1*). This system is widely used for optimal decision-making in the repair of incisional hernias. While this classification is applicable to open hernia surgery, its relevance to minimally invasive surgery (MIS) is less clear as the risk of surgical-site complications is decreased for MIS techniques^14^.

**Table 1:** Modified Ventral Hernia Working Group^13^ classification system for predicting complications after hernia surgery

Grade 1—low risk	Grade 2—co-morbid	Grade 3—contaminated
No history of infection	Smoking Obesity Diabetes Chronic obstructive pulmonary disease Immunosuppression	A: Clean-contaminated B: Contaminated C: Active infection

The HPW classification was described by Petro and Novitsky^15^ in 2016 as a TNM-like classification for ventral hernias. The hernia (H) is staged based on defect width alone: H1, less than 10 cm; H2, 10–20 cm; and H3, greater than 20 cm. The patient (P) is staged on whether there is no co-morbidity (P0) or the presence of morbid obesity (BMI greater than 35 kg/m^2^), smoking, diabetes, and/or immunosuppression (P1). The wound (W) is staged as clean (W0) or contaminated (W1). The HPW stage gives an estimate of postoperative complications and recurrence risk. For example, HPW stage 4 (H3 P1 W1) leads to a 39% risk of short-term complications and a 31% risk of recurrence, whereas HPW stage 1 (H1 P0 W0) poses a 6% risk of short-term complications and a 5% risk of recurrence^15,16^ (*Table 2*).

**Table 2:** Hernia-Patient-Wound^15^ classification system for predicting complications after hernia surgery

Stage	Hernia	Patient	Wound	HPW stage	Surgical site events rate (%)	Recurrence rate (%)
1	1	0	0	H1 P0 W0	5.8	4.7
2	1 or 2	Any	0	H1 P1 W0 H2 Any W0	12.6	9.7
3	Any	Any	Any	H1 Any W1 H2 Any W1 H3 Any W0	20.2	13.2
4	3	Any	Any	H3 P1 W0 H3 Any W1	38.9	31.1

Hernia defect width: H1, <10 cm; H2, 10–20 cm; and H3, >20 cm. Patient co-morbidity: P0, no co-morbidity; and P1, morbid obesity, smoking, and/or immunosuppression. Wound status: W0, clean; and W1, contaminated.

The CeDAR tool is a mathematical equation that predicts the risk of postoperative complications and the associated costs. In the CeDAR app, a surgeon can calculate a specific patient’s risk by entering patient- and procedure-related characteristics and in that way easily show the patient how smoking cessation or weight loss for instance would decrease the risk of complication^17^.

### Definition of complex ventral and incisional hernia

The complexity of a ventral or incisional hernia is affected by many different factors related to the patient, the hernia, and previous abdominal wall surgeries. There is no simple definition or classification system to define a complex hernia.

In 2014, a group of experts agreed on 22 variables to define a complex abdominal wall hernia^18^. These 22 variables were divided into four categories: ‘size and location’ (defect width greater than 10 cm and loss of domain), ‘contamination/soft tissue condition’ (such as wound ulcers, skin graft, and previous open abdomen), ‘patient history/risk factors’ (such as obesity, diabetes, and steroid use) and ‘clinical scenario’ (such as emergency repair and previous mesh repair). Further, patient groups were subgrouped based on complexity into ‘minor’, ‘moderate’, and ‘severe’, depending on the number and type of variables present.

A recently published Delphi consensus endorsed by the EHS agreed on 18 variables, where the presence of one or more of these variables defined a complex abdominal wall hernia with no further subgrouping on grade of complexity^19^. These were divided into four categories: ‘hernia-related variables’ (width greater than 10 cm, recurrent incisional hernia, loss of domain, and midline with concomitant parastomal hernia), ‘operation site-related variables’ (skin defect, fistula, mesh infection, abdominal wall infection, stoma presence, and previous open abdomen), ‘abdominal wall-related variables’ (previous anterior or posterior component separation, previous transverse rectus abdominis myocutaneous flap, previous abdominal wall resection, and hernia after bone resection), and ‘patient-related variables’ (BMI greater than 40 kg/m^2^ and cirrhosis with ascites).

### Loss of domain

Loss of domain is a term used to describe the distribution of abdominal content between the hernia sac and the abdominopelvic cavity. Often, with a large hernia sac, the abdominal contents lie permanently outside the abdominal cavity and it is not possible to reduce all the contents. This may be due to loss of volume of the abdominal cavity and the enlargement of the hernia orifice that occurs due to contraction of lateral muscles. With loss of domain, it can be challenging to close the fascia and significant physiological difficulties may occur when the contents of the hernia sac are reduced back into the abdominal cavity during surgery, which in the worst case scenario can lead to abdominal compartment syndrome or pulmonary failure^20^.

Volumetric measurements and mathematical models are available for assessing loss of domain by CT or other radiographic imaging, by calculating the ratio between the volumes of the hernia sac and the abdominopelvic cavity. The Sabbagh method defines loss of domain as present when greater than or equal to 20% of the total peritoneal volume is in the hernia sac and the Tanaka method defines loss of domain as present when the ratio between the volumes of the hernia sac and the abdominopelvic cavity is greater than or equal to 0.25^21,22^.

In 2020, a group of experts proposed a new definition for loss of domain using an international Delphi consensus process: ‘A ventral hernia large enough such that simple reduction in its contents and primary fascial contents and primary fascial closure cannot be achieved without significant risk of complications due to the raised intra-abdominal pressure’^20^.

## Mesh planes and characteristics

### Mesh planes

**Fig. 3:**
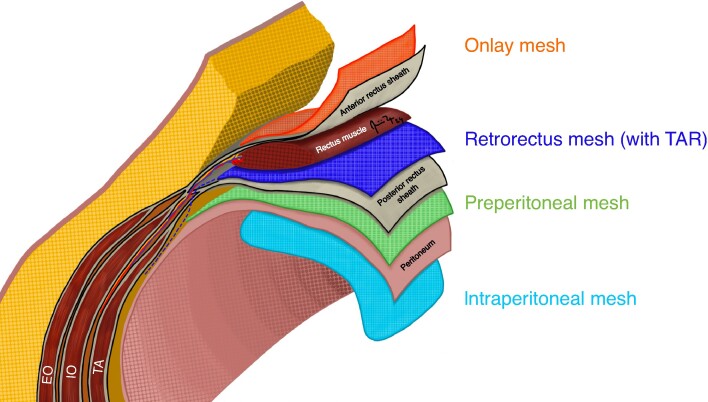
Most used mesh planes EO, external oblique; IO, internal oblique; TA, transversus abdominis; TAR, transversus abdominis release. Artist: Dr Mário R. Gonçalves.

With the advent of many new hernia repair techniques, there is a need for standardization of terminology. A variety of nomenclature for the descriptions of mesh planes has been used in the literature and terms such as ‘sublay’ and ‘underlay’ are non-specific and can refer to different anatomic planes. In 2020, a Delphi consensus was performed by 20 international experts to standardize mesh terminology^23^. The proposed terms for each abdominal wall plane are: onlay (on the anterior fascia), anterectus (between the anterior rectus sheath and the rectus muscle), inlay (attached to edges of hernia defect with no overlap), interoblique (between the internal and external oblique muscles), retro-oblique (below the internal oblique muscle and above the transversus abdominis muscle), retrorectus (between the rectus muscle and the posterior rectus sheath), retromuscular after transversus abdominis release (TAR) (medial as retrorectus and lateral between the transversus abdominal muscle and the transversalis fascia), transversalis fascial (between the posterior rectus sheath and the transversus abdominis muscle), preperitoneal (between the transversalis fascia and the peritoneum), and intraperitoneal (between the peritoneum and the abdominal cavity)^23^. This classification system is thorough and is more specific to the anatomy of the abdominal wall. Despite the more complex terminology, many hernia surgeons most commonly use the onlay, retrorectus, retromuscular after TAR, preperitoneal, and intraperitoneal planes (*Fig. 3*).

### Mesh types

There are many different mesh products available and it is helpful to place them in broader categories based on their composition. It has been proposed that standardized mesh package labels be created utilizing the following terminology^24^: synthetic, biological, bioabsorbable or synthetic absorbable, and hybrid. Each mesh can also be described using characteristics including pore size, mesh weight, and material.

Two of the most used characteristics to describe synthetic meshes are mesh weight and pore size. Meshes can be categorized by weight (ultra-lightweight, less than 35 g/m^2^; lightweight, equal or greater than 35–50 g/m^2^; medium weight, 51–90 g/m^2^; and heavyweight, greater than 91 g/m^2^) and pore size (microporous, less than 100 µm; small pore, 101–600 µm; medium pore, 601–1000 µm; large pore, 1001–2000 µm; and very large pore, greater than 2001 µm)^25^. While there is no conclusive evidence on how these features may impact hernia repair outcomes, the current literature supports the use of large pore, medium weight meshes. This recommendation is thought to be related to improved tissue ingrowth and decreased foreign body sensations of patients^26^ and this viewpoint is also supported by a recent systematic review that reported heavyweight mesh resulting in patients experiencing more foreign body sensations than medium weight and lightweight meshes and having higher rates of seroma formation in retrorectus repairs^27^. Although mesh choice should be individualized for each hernia repair based on hernia and patient characteristics, in clean surgical cases, permanent synthetic mesh is the most used material and is the current standard for hernia repair. There remains controversy about the type of mesh in clean-contaminated and contaminated cases, but permanent synthetic mesh also appears to be a viable option, although long-term data on outcomes in this patient population are not well studied^28,29^.

Synthetic mesh has been the most commonly used mesh material since it was first described by Usher *et al*.^30^ in 1959. The most used synthetic mesh materials are constructed with polypropylene, polyester, polyvinylidene fluoride (PVDF), or expanded polytetrafluoroethylene (ePTFE). While there are potential advantages and disadvantages with each of these different materials, permanent synthetic meshes have been the most widely used and studied materials for hernia repair. Despite their proven safety and efficacy, there has been concern among many surgeons regarding the use of permanent synthetic meshes in contaminated cases. Therefore, biological meshes were developed. These meshes are made from biological tissues, including human and animal donors, and are thought to be less prone to (chronic) infection. Trials on contaminated cases are difficult to perform as patients are heterogeneous and there are limited high-quality data available in clear favour of either synthetic or biological meshes^31^. Nevertheless, the Ventral Hernia Working Group suggested the use of a non-synthetic mesh in contaminated cases^12^.

Bioabsorbable or synthetic absorbable mesh has recently emerged on the mesh scene as a cheaper alternative to biological meshes^32^. This group of mesh products consists of different resorbable substances, which are mostly synthetic and sometimes biological, with resorption times ranging from months to around 1.5 years. As such mesh is fairly new, there is no long-term data comparing bioabsorbable or synthetic absorbable mesh with biological or synthetic mesh. The newest mesh type available is hybrid mesh, which is a combination of biological and permanent synthetic meshes; this mesh is very new to the market and efficacy data are currently unavailable^33^.

## Preoperative optimization

**Fig. 4:**
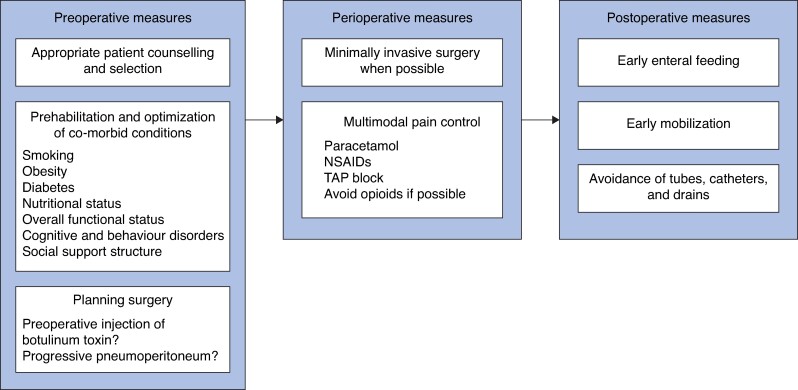
Overview of preoperative, perioperative, and postoperative measures suggested to improve outcomes after ventral hernia repair in an enhanced recovery protocol NSAIDs, non-steroidal anti-inflammatory drugs; TAP, transversus abdominis plane.

For patients needing elective hernia repair, efforts should be made to optimize modifiable patient- and hernia-related characteristics to improve outcomes without unacceptably delaying surgery^34^. As mentioned in the hernia classification systems for predicting complications, some specific factors have a high impact on the risk of complications (*Fig. 4*).

### Smoking cessation

Smoking cessation for at least 4 weeks before surgery is recommended for all types of primary and incisional hernia repair, as it decreases the risk of wound and pulmonary complications^35,36^.

### Weight loss

Obesity poses a specific problem for abdominal wall hernia repair, as it increases the risk of wound complications and recurrence^4^. The overall complication rate is higher in patients with a BMI greater than 30 kg/m^2^ and there is a linear relationship between surgical-site complications and obesity^37^. Surgeons should be cautious about offering patients with a BMI greater than 35 kg/m^2^ elective hernia surgery before attempting weight loss. But these recommendations are currently being challenged; with the advent of MIS techniques, it may be possible that the prior wound morbidity seen in these patients will dramatically decrease^31,38^.

Lifestyle measures alone often do not yield good results. The availability of glucagon-like-peptide-1 receptor agonists and their beneficial effects on weight loss have opened new management pathways that may help obese patients lose weight in 3 to 6 months before hernia surgery. Bariatric surgery is possible and safe in obese patients with complex ventral hernias^39^. Concomitant hernia repair with weight loss surgery is also an option.

### Glycaemic control

Patients with poorly controlled diabetes before surgery have an increased risk of postoperative wound complications and, therefore, hernia recurrence^35,40^. Although one study on ventral hernia repair reported no association between glycated haemoglobin levels and adverse outcomes^41^, it is suggested to evaluate whether glycaemic control, aiming for a preoperative glycated haemoglobin below 8%, can be achieved and to implement monitoring and correction of blood glucose levels throughout the perioperative interval.

### Prehabilitation

Often, patients report that their hernia limits their ability to perform physical exercise or they are afraid that exercise may make their hernia worse. Only a few studies have evaluated whether preoperative exercise influences postoperative outcomes after ventral hernia repair and it seems that improved functional status has a positive impact on postoperative outcomes^42–45^. However, counselling of patients takes time and it may be challenging for surgeons to guide patients in exercise regimens, though an encouragement of improving functional status in some way is suggested.

### Preoperative injection of botulinum toxin

For patients with complex hernias with loss of domain, the application of botulinum toxin before surgery can be used for facilitating fascial closure. With the use of ultrasonographic guidance, botulinum toxin A (BTA) may be injected into the oblique abdominal wall musculature, which leads to relaxation and elongation of the muscles with the aim of achieving fascial closure and potentially decreasing the need for myofascial releases. The majority of published studies used onabotulinumtoxin A (Botox^®^, Allergan, Dublin, Ireland) with a total administered dose varying between 100 and 300 units, divided into three injections bilaterally, and administered on the anterior axillary line, between the costal border and iliac spine, 4 weeks before surgery^46^. Complications are sparse and minor, limited to haematoma and pain at injection sites. While there has been increasing use of this modality in complex hernias, evidence regarding standardization of patient selection, injection techniques, and regimens is still emerging^46–48^.

### Progressive pneumoperitoneum

Progressive pneumoperitoneum (PPP) may be used as an alternative to or in conjunction with the injection of botulinum toxin for patients with complex ventral hernias with loss of domain. The idea behind PPP is an intermittent insufflation to gradually stretch the contracted abdominal wall muscles. A PPP catheter is inserted into the peritoneal cavity and then 800–1000 ml of gas is insufflated daily using a syringe, dictated by patient tolerance (abdominal wall distension and dyspnoea)^49^. There is no consensus in the literature about the type and amount of gas that must be administered or the frequency and duration of insufflation. But complications such as pulmonary dysfunction, thromboembolic events, bowel injury, and even one fatality have been described^50,51^.

## Umbilical and epigastric hernias

Umbilical and epigastric hernias are two of the most common ventral hernias. The true incidence of umbilical and epigastric hernia is unknown, but repairs are frequently performed^52^. Patients with umbilical and epigastric hernias may be asymptomatic or present with complaints such as a bulge, intermittent pain, and discomfort. Non-operative management of an umbilical or epigastric hernia with a watchful waiting approach is safe, although around 20% of patients may ultimately go on to have surgery^53^.

Most patients with a primary midline hernia do not require imaging for diagnostic reasons. However, in patients with large hernias, potential loss of domain, obesity, possible rectus diastasis, unclear defect size, or clinical suspicion of additional defects, imaging with either ultrasonography or CT is recommended for planning the right surgical approach^10^.

Operative strategies may include open repair or a minimally invasive approach, depending on patient characteristics, the size and position of the defect, and cost concerns, as well as the surgeon’s skills and the available equipment^54^.

For the smallest defects (less than 1 cm), the current consensus favours an open repair technique. The decision between suture and mesh repair should be tailored to each case, considering factors such as patient preferences, among others. Mesh seems to decrease the risk of recurrence, even in the smallest primary hernias, at the cost of a slightly increased risk of wound complication^55–57^. When a mesh is used, a flat preperitoneal polypropylene mesh is advised with a recommended overlap of 2 cm^10,58,59^. This plane preference is based on the demonstrated efficacy of this method in reducing recurrence rates, along with its relative simplicity and low complication profile.

As defect size increases to the medium range (1–4 cm), a strong recommendation for mesh use is emphasized, increasing the mesh overlap to 3 cm for open repairs to reduce recurrence, while minimally impacting wound complications and postoperative discomfort^10^. The choice between open and MIS techniques depends on several factors, including patient-specific conditions and the surgeon's expertise, with MIS being considered for patients at a higher risk of wound complications or those with multiple defects^10,54,60,61^. MIS has the added advantage of identifying occult multiple primary hernias and placing a larger mesh in patients with a weak linea alba in the presence of a rectus diastasis. Primary midline hernias with defects greater than 4 cm are rare and they are suggested to be treated as incisional hernias^10^.

## Incisional hernias

Of all ventral hernias, incisional hernias have the most heterogeneous presentation in terms of size, location, and associated complexities. Risk factors for the development of incisional hernias include patient-related factors, pathology at the time of surgical intervention, incision location, and technical factors regarding the laparotomy closure^62^.

Cross-sectional CT imaging for evaluation of incisional hernias is a common practice for the purposes of surgical planning, anatomical identification, and identification of prior mesh (when applicable), as well as the exclusion of perpetuating risk factors such as ongoing sepsis and space-occupying abdominal pathology^35^. When the defect width is greater than 8 cm or there is loss of domain, it is likely that the fascia may not be closed without a myofascial release, which is important information for surgical planning^35^. Furthermore, the defect’s proximity to bony structures is another factor that needs to be considered, as defect closure and sufficient mesh overlap may be difficult or mesh fixation with bone anchors may be necessary.

Patients with reducible asymptomatic incisional hernias have a low risk of emergency repair and may be managed without surgery^63^. However, incisional hernia defects tend to grow larger over time^64^. Patients with symptomatic incisional hernias affecting their quality of life should be offered surgical repair when they are medically fit for surgery^35^. For patients whose quality of life may not be improved with surgery, discussions of potential complications and modifiable risk factors should be done before surgery.

As anatomical understanding of the abdominal wall has evolved, prior open reconstructive techniques have been adapted and the standard of care for open surgical techniques has changed. The retrorectus dissection and retromuscular position for mesh is widely considered the most robust of open surgical techniques and is the starting point for a variety of abdominal wall reconstruction techniques of the midline^35^. However, open repair poses a significant risk of postoperative wound complications and it is often necessary for the patient to stay in hospital for some days after surgery^65^.

MIS techniques have continued to gain popularity for the reconstruction of both midline and non-midline incisional hernias. The intraperitoneal onlay mesh (IPOM) technique with defect closure and appropriate mesh fixation strategies is a surgical technique with many decades of data to support its adoption^66–68^. However, fear of intra-abdominal adhesions and risk of pain caused by traumatic mesh fixation have led to the development of newer MIS techniques with placement of the mesh in the preperitoneal or retrorectus plane^69–72^. Different approaches are available and may be done with regular laparoscopic equipment or with a robotic platform. These techniques are technically demanding and can be relatively long operative procedures, but, because postoperative pain, duration of hospital stay, and the risk of surgical-site infections are reduced, they continue to be a compelling choice for the advanced minimally invasive surgeon.

## Spigelian hernias

A Spigelian hernia represents a rare occurrence of a primary, non-midline hernia, typically characterized by a defect within the Spigelian fascia—an anatomical structure defined as the aponeurosis bounded by the linea semilunaris laterally and the rectus abdominis muscle medially. Predominantly found below the arcuate line where the posterior fascia of the rectus abdominis is missing, these hernias can also manifest above the arcuate line in up to 10% of cases^73^.

Spigelian hernias are often asymptomatic, but may present as an emergency with an irreducible lump, bowel obstruction, or septic complications. While ultrasonographic imaging has demonstrated utility, particularly for specific demographics such as pregnant patients and children, CT imaging offers superior anatomical delineation of the abdominal wall and its visceral associations.

Specific data on watchful waiting or comparative data on operative *versus* non-operative management of Spigelian hernias do not exist. Ultimately, the decision to operate on asymptomatic Spigelian hernias should be made on a case-by-case basis, considering factors such as the size and location of the hernia, as well as the patient’s overall health and medical history, risk tolerance, and preferences. The balance between avoiding an emergency presentation and overtreatment of a relatively benign condition remains a matter for the informed consent process^73,74^.

Repair with a preperitoneal mesh is the simplest surgical technique, which may be performed using an open approach or an MIS approach such as the transabdominal preperitoneal (TAPP) technique or a totally extraperitoneal (TEP) approach for more caudal hernias^75,76^. The specific anatomical location bridging between the triple-layered lateral compartments and single-layered medial compartment makes retromuscular techniques more complex and less favourable.

## Surgical techniques

Abdominal wall reconstruction is unique compared with other types of surgery as there is significant patient heterogeneity in terms of hernia characteristics (that is size, location, etc.), patient anatomy, patient co-morbidity, prior surgical history (recurrence, prior mesh, infection, fistula, stoma, etc.), patient desired goals/outcomes, and surgeon resources. All of these can play a role in determining the best operative strategy for a particular patient. In the constantly evolving field of hernia repair, the choice between open and MIS techniques remains a key decision point. The advent of MIS, particularly laparoscopic surgery, has introduced a paradigm shift, offering the potential to reduce surgical-site infections and shorten recovery time^77,78^. However, these benefits may be overshadowed by technical difficulty, higher costs, and conversion to open surgery^79^. In this section, an overview of the surgical techniques available for primary ventral and incisional hernia repair will be provided, bringing together the benefits and drawbacks of open and minimally invasive approaches to provide surgeons with the necessary knowledge to approach complex abdominal wall reconstruction and choose wisely the most appropriate surgical technique for each case.

### Open techniques

Open surgical approaches are traditionally reserved for larger or more complex hernias, especially those involving significant scar tissue, or when specific patient-related factors or previous surgeries might increase the risk of complications in a minimally invasive procedure. However, for small defects, an open technique is also useful. The choice between suture and mesh repair should be tailored to the specifics of each case, given the limited evidence available.

#### Open preperitoneal technique with mesh for small defects

**Fig. 5:**
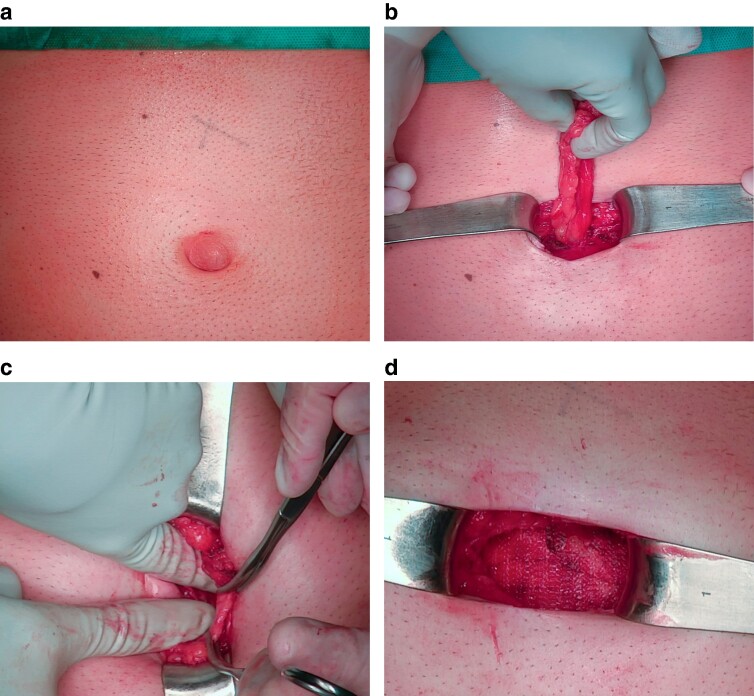
Open preperitoneal technique with mesh for small defects **a** Small umbilical hernia. **b** Sac dissection and exposure of the fascial defect. **c** Preperitoneal space dissection. **d** Mesh positioning (zoomed in). Images courtesy of Dr Amedeo Trippel.

1—Skin incision: perform the incision along Langer’s lines or existing scar lines over the marked hernia site, taking care to prevent inadvertent opening of the hernia sac.
2—Hernia sac dissection: carefully dissect the hernia sac to expose the fascial defect, then proceed to separate the sac from the fascial defect to access the preperitoneal space, using Kocher or Allis clamps as needed (*Fig. 5a*,*b*).
3—Preperitoneal space creation: elevate the fascia and gently extend the preperitoneal space using blunt dissection; adjust the size of the space based on hernia dimensions and required mesh overlap, closing any inadvertent openings in the hernia sac or peritoneum with an absorbable suture; and ensure haemostasis (*Fig. 5c*).
4—Mesh positioning: it is advised to use a macroporous permanent synthetic mesh; and after customizing the mesh size, place it in the preperitoneal space, ensuring it lies centrally beneath the fascial defect (*Fig. 5d*).
5—Mesh fixation: mesh fixation is often not needed, but, when perceived necessary, absorbable sutures should be used.
6—Fascial closure: close the fascia with a slow-absorbing monofilament suture, checking for haemostasis.
7—Skin closure: close the skin according to the surgeon’s/patient’s preference.

#### Rives-Stoppa repair and peritoneal flap repair

The Rives-Stoppa repair with retrorectus mesh placement is considered a standard technique for the treatment of larger primary ventral and incisional hernias, where achieving tension-free fascial closure poses a challenge^80,81^. Despite several modifications over time, the underlying principle involves an extensive dissection of the retromuscular space to allow for a wide mesh overlap beyond the hernia margins, which has been associated with decreased recurrence rates^82,83^. In this procedure, bilateral dissection of the posterior rectus sheath is performed, enhancing fascial closure with a mesh inserted in the retromuscular plane.

The peritoneal flap hernioplasty is a variant of the Rives-Stoppa repair that employs the hernial sac to bridge the fascial gap. This technique helps isolate the mesh from intraperitoneal contents, especially in larger abdominal wall defects, and applies to both midline and transverse hernias, offering a versatile and effective solution with low complication and recurrence rates^84–87^.

#### Rives-Stoppa repair

**Fig. 6:**
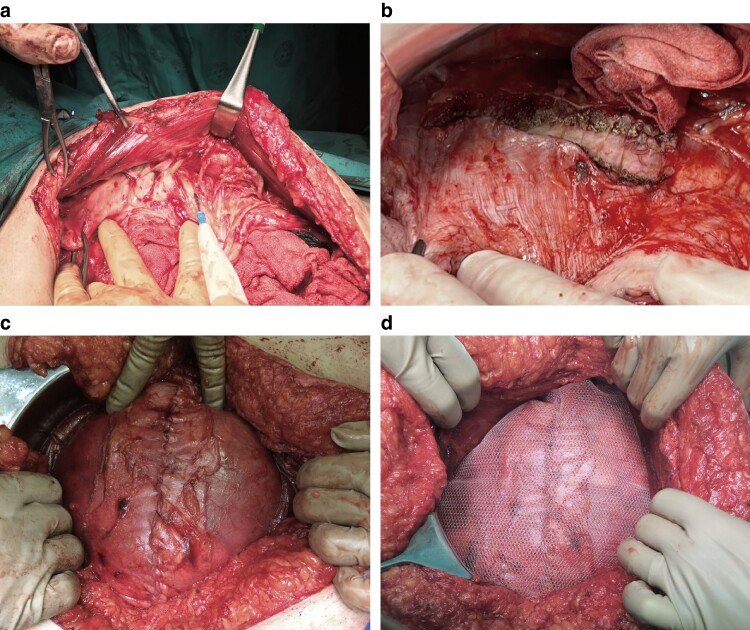
Rives-Stoppa repair **a** Dissection of the posterior rectus sheath. **b** Extended dissection via transversus abdominis release. **c** Closure of the posterior rectus sheath. **d** Positioning of retrorectus mesh. Images courtesy of Dr Heather Bougard and Dr Mário R. Gonçalves.

1—Skin incision: perform an incision based on hernia size for primary ventral hernias or along previous scars for incisional hernias, removing any redundant skin or old scar tissue to ensure proper exposure and enhance cosmetic results.
2—Hernia sac dissection and opening: dissect the hernia sac from the abdominal wall to reveal the fascial defect; and open the sac, carefully separating peritoneal adhesions to prevent damage to internal organs.
3—Adhesiolysis: thoroughly release adhesions between the viscera and the hernia sac, as well as around the defect margins, using both blunt and sharp techniques.
4—Hernia sac excision: when the hernia sac is not needed for closure, excise it along the fascial edges bilaterally.
5—Posterior rectus sheath incision: incise the posterior rectus sheaths bilaterally parallel and about 0.5–1 cm from the fascial edge and separate them from the rectus muscles laterally to the linea semilunaris, avoiding injury to neurovascular bundles. Use Allis or Kocher forceps for traction (*Fig. 6a*,*b*).
6—Caudal dissection: continue preperitoneal dissection downward to Cooper’s ligaments (Retzius space) and connect the preperitoneal spaces across the midline, preserving caudal linea alba integrity.
7—Cranial dissection: continue preperitoneal dissection upward to and below the xiphoid process; and enter the preperitoneal space through the posterior rectus sheath and connect the retromuscular and preperitoneal spaces across the midline, preserving linea alba integrity.
8—Haemostasis: ensure bleeding control from medial perforating vessels during retrorectus dissection.
9—Posterior rectus sheath closure: close the posterior sheath tension-free using a slowly absorbable monofilament suture; repair any fascial perforations; and when tension-free closure of the posterior rectus sheath is not possible, the hernia sac or omentum can be used (*Fig. 6c*).
10—Mesh positioning: place a macroporous permanent synthetic mesh in the retrorectus space, ensuring a minimum 5 cm overlap in all directions (*Fig. 6d*).
11—Mesh fixation: the mesh may be fixed to Cooper’s ligament bilaterally and to the midline or the xiphoid cranially with absorbable sutures, and additional absorbable sutures can be added laterally as needed for secure placement.
12—Drain placement: drains are often not necessary, but may be placed in the retrorectus space above the mesh or subcutaneously when a large space remains.
13—Fascial closure: close the anterior rectus sheath with a slowly absorbable monofilament suture.
14—Skin closure: close skin and subcutaneous layers with suitable sutures, followed by wound care.

#### Peritoneal flap repair

**Fig. 7:**
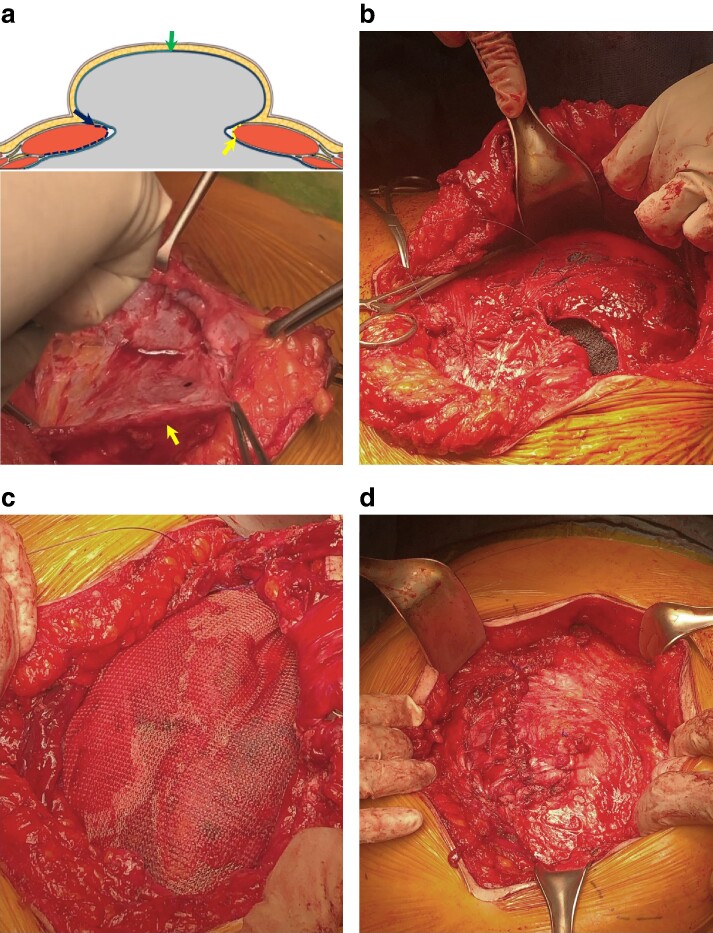
Peritoneal flap repair **a** The schematic provides an axial view of the abdominal wall, highlighting a giant ventral hernia (the arrow at the top of the schematic indicates the hernia sac opening in the middle, the arrow and broken line on the left-hand side of the schematic show the anterior rectus sheath incision site and dissection of the posterior rectus sheath for posterior flap mobilization, and the arrow and broken line on the right-hand side of the schematic show the posterior rectus sheath incision site and dissection towards the anterior rectus sheath for anterior flap mobilization), and the image below the schematic illustrates the incision of the posterior rectus sheath to mobilize the anterior flap on the left side. **b** Closure of the posterior layer incorporating the posterior peritoneal flap. **c** Placement of the retrorectus mesh. **d** Closure of the anterior layer incorporating the anterior peritoneal flap. Images courtesy of Dr Ritu Khare.

Steps 1–3: follow the Rives-Stoppa technique; and it is important to longitudinally open the hernia sac along its entire length (*Fig. 7a*).
4—Posterior flap mobilization: incise the anterior rectus sheath on one side, parallel and about 0.5 cm from the rectus muscle’s medial edge; dissect the 0.5 cm medial border of the anterior sheath and continue between the rectus abdominis and the posterior rectus sheath to access the retromuscular space; and this mobilized flap, now connected to the posterior sheath, forms the repair’s deep (posterior) layer.
5—Anterior flap mobilization: on the contralateral side, similarly incise and dissect the posterior rectus sheath, aligning it with the medial border to extend to the anterior sheath; and this newly created flap serves as the repair’s superficial (anterior) layer (*Fig. 7a*).
6—Retrorectus dissection: develop the retrorectus space bilaterally to the linea semilunaris, avoiding damage to the lateral neurovascular bundles; and use Allis or Kocher forceps for posterior sheath traction.

Steps 7–9: caudal dissection, cranial dissection, and haemostasis as in the Rives-Stoppa technique.

10—Deep/posterior layer closure: suture the posterior peritoneal flap to the contralateral posterior rectus sheath, creating a unified layer across the midline into both retrorectus spaces (*Fig. 7b*).
  Steps 11–12: mesh fixation and drain placement if necessary, as in the Rives-Stoppa technique (*Fig. 7c*).
13—Superficial/anterior layer closure: suture the anterior peritoneal flap to the contralateral anterior sheath’s edge, segregating the mesh from the subcutaneous layer and ensuring a tension-minimized closure (*Fig. 7d*).
14—Skin closure: close the skin and subcutaneous layers with suitable sutures, followed by wound care.

### Minimally invasive techniques with intraperitoneal mesh placement

#### Intraperitoneal onlay mesh technique

The minimally invasive IPOM technique was described in the early 1990s and represented the first minimally invasive strategy for the repair of ventral and incisional hernias, featuring the placement of mesh within the peritoneal cavity to overlay the hernia defect internally^88^. The IPOM technique quickly gained popularity as it was easy to learn with a decrease in wound complication rates compared with open repair^65,68^ and can be done either laparoscopically or robotically.

An advanced modification, known as the IPOM plus technique, incorporates an initial closure of the hernia defect before mesh placement, aiming to restore the abdominal wall’s anatomy, avoid bridging of the mesh, and, therefore, reduce the risk of seroma development and recurrence^89^. Techniques such as laparoscopic intracorporeal rectus aponeuroplasty (LIRA) and IPOM with peritoneal bridging share this core principle^90,91^. In these procedures, the mesh is typically anchored using sutures, tacks, or adhesives, ensuring its fixation and preventing displacement or wrinkling^92–94^.

#### Intraperitoneal onlay mesh plus technique

**Fig. 8:**
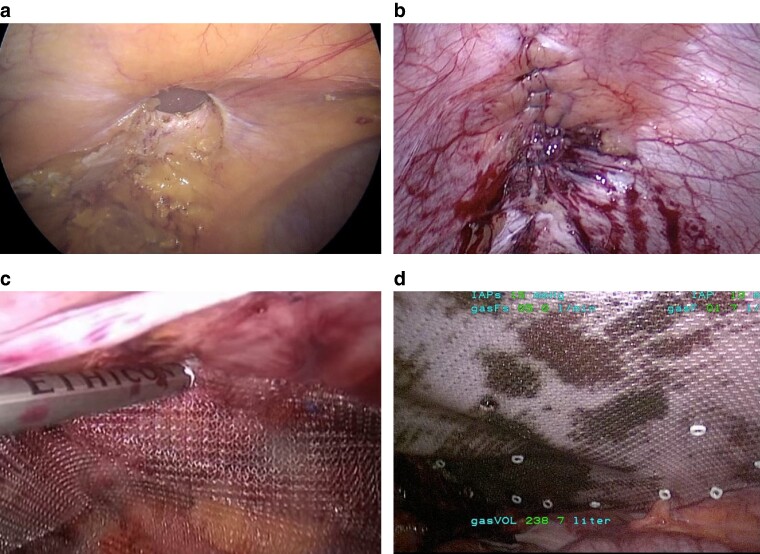
Intraperitoneal onlay mesh plus technique **a** Visualization of the abdominal wall defect after the reduction of hernia contents. **b** Fascial closure. **c** Positioning of the mesh. **d** Intraperitoneal mesh properly extended and fixed. Images courtesy of Dr Heather Bougard.

1—Pneumoperitoneum and first trocar insertion: establish pneumoperitoneum based on the surgeon’s preference; and select an entry point avoiding scars, sites previously operated on, and vascular anatomy to minimize injury risk, typically placing the first trocar lateral to the semilunar line.
2—Additional port placement: the number and position of additional ports are dictated by the hernia’s size and location, and should be incorporated into the surgical planning; anticipate the need for larger or additional trocars for mesh and fixation devices; and, typically, two additional ports are inserted to achieve optimal triangulation.
3—Adhesiolysis and hernia content reduction: carefully separate visceral adhesions to reveal defects in the abdominal wall and prepare for mesh placement; this may include excising excess fatty tissue and ligaments; caution is paramount to avoid direct or thermal damage to internal organs; and confirm complete reduction of hernia contents (*Fig. 8a*).
4—Defect closure: once the hernia defect is fully exposed, the fascia is closed using techniques such as a transcutaneous transfascial interrupted suture or an intracorporeal running or interrupted suture (*Fig. 8b*).
5—Mesh placement preparation: verify the defect size and select a mesh that allows for at least 5 cm overlap; and the chosen mesh should have a parietal side facing the abdominal wall and an abdominal side with anti-adhesive coating facing the viscera (*Fig. 8c*).
6—Mesh insertion and positioning: introduce the mesh via a lateral trocar using graspers; and carefully unfold the mesh to cover the defect, ensuring correct side orientation and overlap.
7—Mesh fixation: secure the mesh with fixation devices, transfascial sutures, or intracorporeal sutures; commonly, initial cardinal fixation is applied, followed by single or double crown techniques; the mesh can also be sewn circumferentially; and reduce intra-abdominal pressure to less than 6 mmHg during fixation, avoiding gaps larger than 1.8 cm (*Fig. 8d*).
8—Trocar site closure: close trocar sites of 10 mm or larger to prevent trocar site hernia; and, in obese patients, using a port-side closure device can help ensure the fascia is properly closed.

### Minimally invasive techniques with extraperitoneal mesh placement

The next few techniques (the TAPP technique, the transabdominal retromuscular umbilical prosthetic (TARUP) technique, and the extended totally extraperitoneal (eTEP) technique) are minimally invasive hernia repair methods that allow for fascial defect closure and extraperitoneal/retrorectus mesh placement (*Table 3*). The repairs differ slightly in terms of the planes of the anterior abdominal wall in which the surgeon is primarily working, access to that space, and appropriateness of certain techniques, depending on hernia size and location. Most of the techniques should be applied for small to medium defects that abide by the bilateral rectus width to hernia defect ratio of greater than 2 : 1 that do not require component separation, although these repairs can be combined with posterior component separation when needed^95^.

**Table 3:** Comparison of different minimally invasive surgery techniques

Technique	Optimal hernia defect size	Hernia location	Mesh placement location	Possible pitfalls/complications
IPOM	Small to medium	Any	Intraperitoneal	Intra-abdominal mesh complications, possible nerve or vascular injury with mesh fixation
TAPP	Small to medium	Any	Preperitoneal	Flap dissection may be difficult in areas with thin peritoneum or scar tissue, hernia sac may not be useful as continuation of the flap
TARUP	Medium or small with concurrent diastasis	Midline	Retrorectus	Potential risk of posterior rectus sheath breakdown, injury to lateral neurovascular bundles
eTEP	Medium or small with concurrent diastasis	Midline	Retrorectus	Small rectus width can decrease feasibility, inability to see intra-abdominally, missed injury to intra-abdominal bowel

IPOM, intraperitoneal onlay mesh; TAPP, transabdominal preperitoneal; TARUP, transabdominal retromuscular umbilical prosthetic; eTEP, extended totally extraperitoneal.

#### Transabdominal preperitoneal technique

A TAPP approach to ventral hernias can be employed in many different situations. Most of the time this approach is done using a robotic platform, although it can be completed laparoscopically^96^. This is one of the most broadly applicable and versatile techniques for minimally invasive hernia repair and can be done for many different hernia locations on the abdominal wall. The goal of the TAPP procedure is to exclude the mesh from the abdominal cavity and is best done in patients who have a robust preperitoneal layer, preperitoneal fat, and non-recurrent hernias. The preperitoneal plane can be more difficult to access in a recurrent incisional hernia or when someone has had prior mesh placement.

**Fig. 9:**
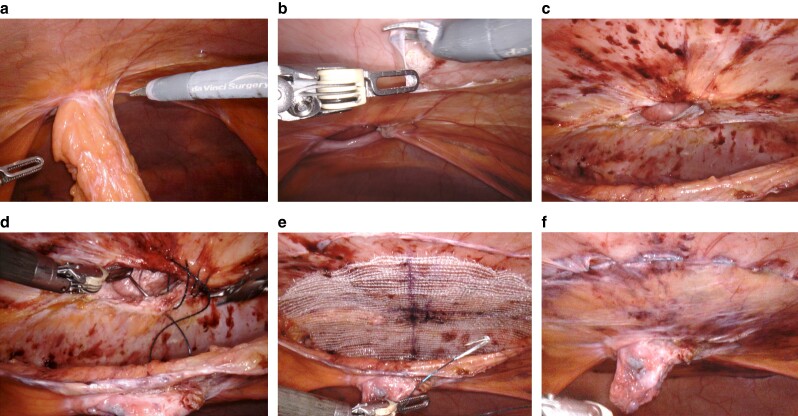
Transabdominal preperitoneal technique **a** Visualization of umbilical hernia with omentum. **b** Ipsilateral peritoneal incision. **c** Overview of peritoneal flap and reduced hernia sac. **d** Defect closure. **e** Mesh placement. **f** Peritoneal flap closure. Images courtesy of Dr Nadia A. Henriksen.

1—Access and trocar placement: this technique consists of abdominal entry and establishment of pneumoperitoneum; once this access is established, additional trocars are placed away from the site of the hernia; and, most often for ventral hernias, the placement of the trocars is in the lateral abdominal wall, but this placement may differ when the hernia is located in the flank, epigastrium, or pubic area (*Fig. 9a*).
2—Preperitoneal flap development: depending on the size of the hernia defect, a preperitoneal flap is developed starting around 5 cm away from the fascial defect of the hernia sac towards the working trocars (*Fig. 9b*); this is carefully dissected away from the posterior rectus sheath of the anterior abdominal wall and the preperitoneal fat is usually taken down with this flap; the key to doing a preperitoneal dissection is to not violate the posterior rectus sheath or linea alba anteriorly; and, once this preperitoneal flap is well developed laterally and around the hernia defect and sac, proceed with hernia sac dissection.
3—Hernia sac dissection: care should be taken to reduce the hernia sac, but, often, the hernia sac can be thin and scarred, and, in the process of reducing it, there can be violation of the sac. When the sac is extremely thin or very large and spiculated, sometimes it will not be possible to reduce it and, in those cases, it must be detached from the fascial defect edges. Once the peritoneal flap is created past the hernia defect, it is extended beyond the hernia itself as much as possible in all directions (*Fig. 9c*). There is no evidence-based guideline regarding preperitoneal flap development size, but as large as feasible to allow for adequate mesh overlap is a general principle.
4—Fascial defect closure: the hernia fascia defect is typically closed with a slowly absorbable suture in a running fashion, taking care to incorporate the anterior fascia (*Fig. 9d*).
5—Mesh placement: in this extraperitoneal plane, a permanent macroporous synthetic non-coated mesh is recommended and should be sized based on the the hernia defect and preperitoneal flap size; this mesh is most commonly placed against the anterior abdominal wall; there are no data on optimal fixation techniques, but most common methods include absorbable sutures placed at key points on the mesh/posterior rectus sheath, fibrin glue, or a combination of both; and, alternatively, a self-fixing mesh can be used (*Fig. 9e*).
6—Preperitoneal flap closure: after the mesh is placed, the extraperitoneal space is closed to exclude the mesh from the intra-abdominal cavity and its contents with a running absorbable suture; and any holes made in the preperitoneal flap during the dissection must also be closed with an absorbable suture to prevent the bowel from becoming incarcerated in the posterior layer or adhered to the exposed mesh (*Fig. 9f*).

#### Transabdominal retromuscular umbilical prosthetic technique

A TARUP hernia repair is the equivalent of a minimally invasive transabdominal Rives-Stoppa repair^72,97^. TARUP was originally described for umbilical hernias, but may be used for epigastric and incisional hernias as well, and may also be referred to as TransAbdominal RetroMuscular (TARM). Unlike the eTEP technique, where the retromuscular space is accessed directly and insufflated without entry into the abdominal cavity, this approach requires access to the abdominal cavity first and then taking down the posterior rectus sheath to gain access to the retromuscular space. This is typically and most easily done for midline ventral hernias. Indications for this procedure include a larger midline ventral defect, an umbilical defect with concurrent diastasis, no existing preperitoneal plane, and recurrent hernias in which the preperitoneal space or intra-abdominal space already has mesh. Because this is a retromuscular repair and technically a minimally invasive Rives-Stoppa operation, there should be caution in using this technique in small ventral defects where a preperitoneal repair may suffice.

**Fig. 10:**
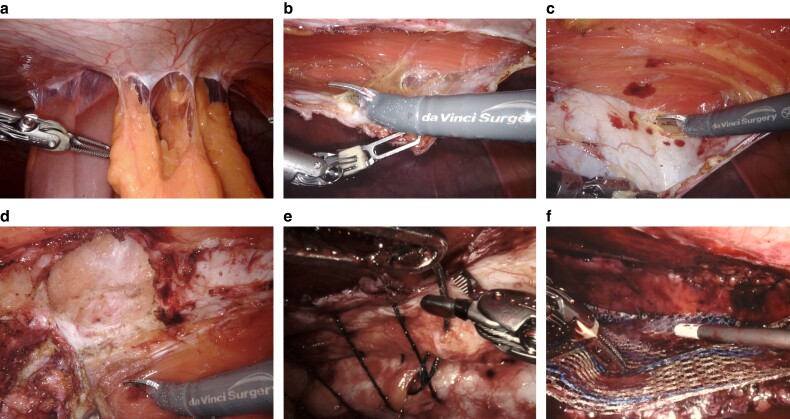
Transabdominal retromuscular umbilical prosthetic technique **a** Visualization of midline hernia with omental adhesions and small bowel. **b** Lateral posterior rectus sheath incision. **c** Medial posterior rectus sheath incision. **d** Midline crossover and hernia sac reduction. **e** Defect closure. **f** Mesh placement. Images courtesy of Dr Nadia A. Henriksen and Dr Jenny Shao.

1—Access and trocar placement: this technique consists of abdominal entry and establishment of pneumoperitoneum; and placement of the trocars lateral to the rectus sheath (*Fig. 10a*).
2—Flap development by lateral posterior sheath incision: development of the retromuscular flap entails incising the lateral border of the posterior rectus sheath starting on the trocar side closest to the surgeon making sure to stay about 1 cm medial of the semilunar line to spare the neurovascular bundles and prevent violation of the semilunar line; and, once the posterior sheath is incised, the rectus muscle fibres are gently swept off the posterior sheath (*Fig. 10b*).
3—Medial posterior sheath incision: once the medial border of the posterior rectus sheath is identified, an incision at least 0.5 cm away from the medial border of the sheath is made to gain access to the preperitoneal space (*Fig. 10c*).
4—Midline crossover: once the medial border of the posterior rectus sheath is incised, access to the preperitoneal space is developed along the midline; this plane is typically initially more easily accessed away from the hernia sac and the epigastrium, where there is more preperitoneal fat, which helps to minimize peritoneal flap defects; when there is diastasis this comes down fairly easily across the midline; and for incisional hernias there can be scarring along the area where the prior incision is (*Fig. 10d*).
5—Hernia sac dissection: similar to TAPP repair, hernia sac reduction should be done when feasible and this is usually easier when the hernia sac is small and is usually harder when the sac is thin or large; and, when not feasible, the sac should be abandoned and the subsequent peritoneal defect should be closed.
6—Contralateral rectus sheath dissection: once the midline crossover is complete and the contralateral posterior rectus sheath is visualized, the medial border of the sheath 0.5–1 cm lateral to the linea alba or midline should be incised to open the posterior sheath; and, once this is done, muscle fibres should be swept off the posterior sheath until the contralateral semilunar line and neurovascular bundles are visualized and spared.
7—Fascial defect closure (*Fig. 10e*), mesh placement (*Fig. 10f*), and flap closure are the same as in TAPP repair (see Steps 4, 5, and 6 in the previous section). Caution should be taken to close the fascial layer on the lateral border to prevent postoperative dehiscence.

Although the flap creation technically differs from that of a TAPP hernia, fascial defect closure, mesh placement, and flap closure are the same. It is worthwhile noting that the same size TAPP flap can be created as a TARUP flap, just in differing planes of the abdominal wall.

#### Extended totally extraperitoneal technique

The eTEP approach builds upon the principles of traditional TEP for inguinal hernias, but extends its application to a wider range of hernias, including ventral and incisional hernias^71,98^. Some of its advantages lie in the direct access to the retromuscular space, reducing the risk of intra-abdominal complications and allowing the use of larger meshes with less need for fixation. Despite the encouraging outcomes associated with the eTEP approach, it requires a high level of surgical skill and familiarity with advanced minimally invasive techniques.

**Fig. 11:**
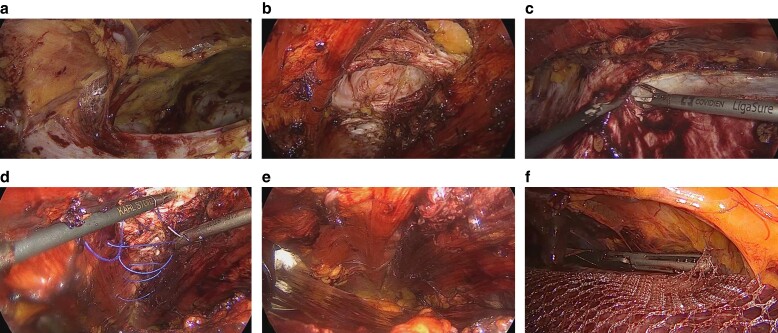
Extended totally extraperitoneal technique for a lower midline defect **a** Visualization of the posterior rectus sheath from the highest left port, with dissection proceeding in a caudal direction. **b** Visualization of the fascial defect after hernia sac dissection. **c** Extended dissection performed via transversus abdominis release. **d** Initial stage of fascial closure. **e** Completion of fascial closure. **f** Mesh positioning. Images courtesy of Dr Anibal Pimentel.

1—Access to the retrorectus space: access is guided by the hernia’s location and prior abdominal surgeries; and, generally, it is best to start developing the retrorectus space away from the hernia and previous surgical sites.
  - For upper midline defects, create a horizontal incision 2 cm below the umbilical line and just medial to the linea semilunaris. Open the anterior sheath and develop the retrorectus space cranially and caudally using a balloon dissector, avoiding overinflation. Alternatively, this can be done using a direct optic technique, instead of using a balloon dissector.
  - For lower midline defects, access the upper left retrorectus space via a 2 cm paramedian horizontal incision, then proceed similarly to develop the space.
2—Additional trocar placement: place extra trocars under direct vision as the retrorectus space is developed.
  - Upper midline defects require at least two additional ports: one 3 cm above the pubic symphysis in the left paramedian line and another 2 cm above it in the right paramedian line.
  - Lower midline defects require two ports: one near Palmer’s point and another near the umbilical line between the left midclavicular and anterior axillary lines.
3—Posterior rectus sheath dissection: use a 30° scope for dissection towards the hernia defect.
  - For upper midline defects: from the lowest port, identify and dissect the posterior rectus sheath on both sides in a cephalad direction, connecting the retrorectus spaces.
  - For lower midline defects: with the scope in the highest port, dissect the caudal posterior rectus sheath until the pubis is visible; then return to the upper abdomen and incise the left posterior rectus sheath near the linea alba, enter the preperitoneal space, and similarly incise the right sheath to access the opposite retrorectus space; and continue dissection and position a fourth port at the level of the initial port on the right paramedian line for bilateral dissection (*Fig. 11a*,*b*).
4—Hernia sac dissection: identify and either peripherally dissect or incise the hernia sac for adhesiolysis before closing the fascia; and consider a TAR for larger defects or when closure is challenging (*Fig. 11c*).
5—Fascial closure: lower pneumoperitoneum to 8 mmHg and close the defect using a slowly absorbable suture in a running fashion, possibly also addressing diastasis recti when present (*Fig. 11d*,*e*).
6—Mesh preparation and positioning: choose a suitable mesh size for the dissected space (a medium weight macroporous permanent synthetic mesh is generally recommended); and insert and position the mesh to adequately cover the defect (*Fig. 11f*).
7—Mesh fixation: although some surgeons opt not to secure the mesh, fixation can be achieved using transfascial sutures, tacks, or adhesives as necessary.
8—Pneumoperitoneum release: gradually release the pneumoperitoneum, ensuring the mesh remains flat and properly extended.

#### Hybrid approach

Sometimes it is possible to utilize a hybrid open and minimally invasive approach to fix an abdominal wall hernia. This can be useful in specific cases where scar revision, excision of the hernia sac, or soft tissue rearrangement is needed and outweighs the risk of wound complications. Occasionally, after a minimally invasive repair, there is not adequate release on the skin and subcutaneous tissue, and a small incision may need to be made to excise the sac and revise the soft tissue plane, especially when the patient is concerned about the cosmesis of the repair.

### Component separation techniques

For larger fascial hernia defects (often defined as greater than 10 cm in width or having a bilateral rectus width to hernia defect ratio of less than 2 : 1), a purely retrorectus or peritoneal flap dissection may not be enough to completely approximate the midline anterior fascia without tension^95^. In these cases, component separation may be required. In brief, component separation allows for sliding the different muscle components of the abdominal wall that can help achieve a tension-free midline anterior rectus sheath closure^99^. There are multiple different types of component separation and the two most utilized techniques are anterior component separation (external oblique release (EOR)) and posterior component separation (TAR). Performing one type of release over the other is a nuanced surgical decision and factors such as hernia characteristics, patient anatomy, other patient factors, and type of hernia repair performed can all impact what type of component separation is appropriate. When experience in these techniques is lacking, a patient who possibly requires a component separation procedure should be referred to a specialist centre.

#### Anterior component separation

Historically, the first technique popularized and widely adopted was described by Ramirez *et al*.^100^ in 1990, namely the incision of the external oblique muscle laterally and the separation of the muscle from its underlying internal oblique and transversus abdominis planes (called EOR or anterior component separation). It is often performed as an addition for a retrorectus dissection and mesh placement. This release provides about 4–8 cm of rectus advancement on either side of the abdominal wall and expands the abdominal cavity to provide appropriate fascial defect closure^101,102^. This technique can be done either as an open prodecure with or without a perforator-sparing technique or endoscopically.

**Fig. 12:**
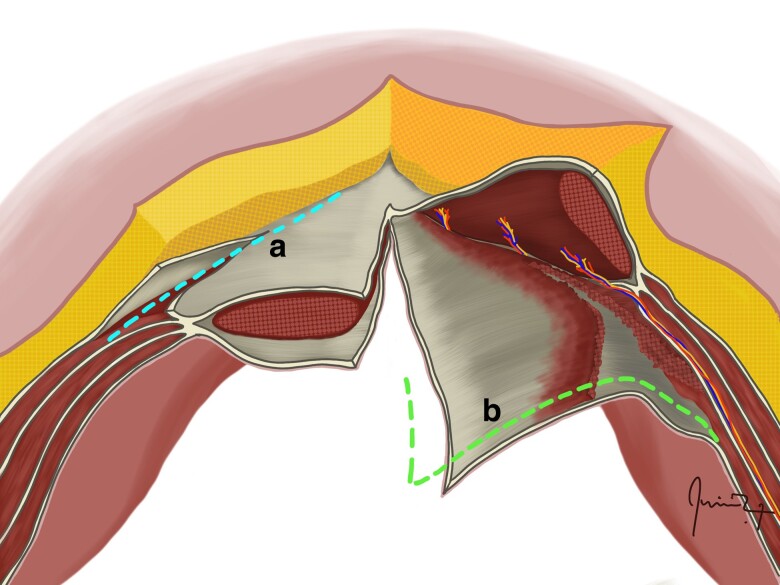
Component separation techniques **a** Anterior component separation (external oblique release). **b** Posterior component separation (transversus abdominis release). Artist: Dr Mário R. Gonçalves.

The external oblique muscle can be accessed by creating a large soft tissue flap and mobilizing the subcutaneous fat from the anterior fascia until the external oblique muscle is visualized beyond the semilunar line (*Fig. 12a*). The muscle is incised lateral to the semilunar line until the internal oblique muscle or the fascial plane between the muscles is visualized. The muscle is divided and afterwards pushed laterally to allow the rectus muscles to slide further medially. This technique is often very effective for large subxiphoid and epigastric hernias, given the location of the fascial release. Retrorectus, intraperitoneal or preperitoneal mesh placement is possible. One of the drawbacks of an open EOR is the risk of wound complications, which can negatively impact hernia repair and have other undesired consequences^103^.

For those reasons, less invasive approaches to EOR have been developed to help decrease these morbidities, while achieving the same fascial release^104,105^. Open EOR can also be done using a perforator-sparing method in which the subcutaneous perforators are spared when exposing the lateral external oblique muscle. For the perforator-sparing technique, the subcutaneous tissue is taken above and below the major perforators to reach the lateral external oblique muscle. Once this is incised, use retractors to elevate and retract the intact subcutaneous tissue to access the muscle laterally. This perforator-sparing option has been shown to decrease potential wound complications in comparison with the traditional open approach^106^.

Another minimally invasive option is to perform an endoscopic EOR, which negates the need for large soft tissue mobilization. Technical aspects of endoscopic EOR were initially described in 2007 and the technique has continued to evolve^107,108^. Using a small lateral incision (2–3 cm) bilaterally over the area of the external oblique muscle, a direct cut down to the external oblique muscle is performed. Often, this is most easily achieved by marking out the boundaries of the rectus and starting high on the insertion of the external oblique muscle on the lower ribcage. Once the muscle fibres are exposed, visual confirmation of the direction of the muscle fibres will confirm the right space directly under the external oblique muscle. Using a trocar, insufflation, and a camera, the space between the external and internal oblique muscle can be bluntly developed. Alternatively, a spacemaker balloon dissector can be placed into this space and insufflated to open the correct plane. Once this is achieved, and the space insufflated, a second lateral trocar can be placed to operate an energy device to divide the external oblique muscle fibres longitudinally. After the division, the space between the external oblique and internal oblique muscle must subsequently be bluntly developed laterally to create a similar release^108^.

#### Posterior component separation

Since the early 2010s, with the popularization of the Rives-Stoppa procedure and retrorectus mesh placement, posterior component separation has been described as an extension of the retrorectus plane. Posterior component separation has taken over as the predominant technique for many abdominal wall surgeons as it allows for fascial release with tension-free repair of large hernia defects, placement of large pieces of mesh in the retromuscular plane, and avoidance of large subcutaneous skin flaps and subsequent wound complications. Described by Novitsky *et al*.^109^ in 2012, the posterior component separation or TAR technique affords advancement of 4–8 cm on either side of the abdominal wall^101,102^.

When done during open ventral hernia repair, the medial border of the posterior rectus sheath about 0.5–1 cm from the linea alba is incised along the midline. The rectus muscle is swept superiorly off the posterior rectus sheath until the lateral border and the neurovascular bundles are identified. Then the posterior rectus fascia and the transversus abdominis muscle along its medial insertion are divided, staying medial to the neurovascular bundles, making sure to preserve the innervation of the abdominal wall and the semilunar line (*Fig. 12b*). Once the posterior sheath and muscle or tendinous transversus abdominis fibres are incised and the correct plane entered, the muscle fibres of the transversus abdominis are then swept upwards off the transversalis fascia and peritoneum below, extending laterally out to the flank. The large space created can extend inferiorly past the arcuate line to the space of Retzius and can connect superiorly to the preperitoneal plane at the falciform ligament. The posterior rectus sheaths from both sides are then closed in the midline using an absorbable suture to exclude the abdominal contents from the mesh. This allows for dramatic extension of the plane for large mesh reinforcement of the entire abdominal wall and provides adequate muscle and fascial release for subsequent midline fascial defect closure.

This technique can also be done as a minimally invasive procedure. Compared with anterior component separation, TAR has little to minimal associated additional wound morbidity and a decreased complication profile, making it increasingly popular among hernia surgeons for larger and more complex ventral wall hernias^110^.

It is worth noting that the anterior component and posterior component separation techniques should not be used concurrently in the same patient as there is a higher likelihood of creating abdominal wall destabilization and potential detachment of the abdominal wall along the semilunar line on one or both sides of the abdomen.

### Intraoperative fascial traction

In recent years, a new method for approximation of large fascial defects has been described with promising results, decreasing the need for component separation^111,112^. A fascial traction device is applied intraoperatively with transfacial sutures and perpendicular traction is performed on the abdominal wall for around 30 min. Intraoperative fascial traction may be used together with preoperative injections of BTA and has been described for both open and MIS procedures^112,113^.

### Use of drains

The role of drains in managing fluid accumulation and potentially reducing seroma and haematoma formation post-hernia repair remains a topic of debate due to a lack of high-quality clinical evidence. While there is widespread discussion in the literature, the evidence from clinical trials is sparse and inconclusive, leaving the benefits of drain usage in ventral hernia repairs uncertain^114–116^.

It seems that drains increase the duration of hospital stay and are associated with a risk of infection without reducing the risk of seroma with certainty^117^. Further research is needed to provide conclusive evidence on the efficacy of drains in hernia surgery. Until then, the decision to use drains may be best determined on a case-by-case basis, emphasizing the need for a personalized approach in the absence of comprehensive evidence.

### Plastic reconstructive techniques

Complex abdominal wall reconstruction is a comprehensive approach to hernia repair and can include considerations of how the soft tissues around the hernia defect interplay with necessary tissue coverage, cosmetic outcomes, function, and quality of life. Patients who undergo excess weight loss before surgery and have a large pannus and a concurrent ventral hernia can present with unique challenges that can be addressed at the time of hernia repair. There are certain advantages to undergoing a concurrent panniculectomy, which include improved exposure, removal of scar and poorly perfused tissue, and improved cosmesis and function, all of which can contribute to the success of a large abdominal wall reconstruction^118^. This has been proven to be feasible without negatively impacting long-term hernia outcomes and should be considered as an option for those patients who meet the criteria^119^. Plastic reconstructive techniques should be performed by a surgeon trained in these techniques.

## Postoperative care and complications

### Enhanced recovery protocols

Enhanced recovery protocols (ERPs) represent a multimodal, multidisciplinary approach to enhance surgical outcomes for minimizing surgical stress and its effects, leading to an improvement in outcomes for patients^120^. ERPs are still not widely implemented in hernia surgery, but a few studies have shown that ERPs in ventral hernia surgery are associated with a decreased duration of hospital stay^121^.

ERPs permeate every aspect of patient care, including preoperative, intraoperative, and postoperative care initiatives (*Fig. 2*). First, MIS has demonstrated important benefits for patients, leading to reduced wound size, complications, and pain, as well as quicker recovery, and is advocated as the preferred technique for ERP implementation in abdominal wall reconstruction^121–123^. Second, multimodal pain control is important and may reduce opioid consumption^120^. Some medications administered before surgery to aid in postoperative pain control, such as paracetamol, ibuprofen, and systemic infusions, including lidocaine or ketamine, as well as local or regional anaesthesia and preoperative blocks, such as the transversus abdominis plane (TAP) block, decrease time to recovery and the duration of hospital stay^121^. A TAP block targets afferent nerves innervating the abdominal wall in the space between the internal oblique and transversus abdominis muscles and may be applied using landmark-guided visualization, ultrasound-guided visualization, or under direct visualization during open surgery or MIS^124,125^.

### Return to activity after surgery

There are no evidence-based recommendations on when to return to work and exercise after ventral hernia surgery. The EHS suggests in the guidelines for the management of midline incisional hernias, in a good clinical practice statement, that patients should be encouraged to actively mobilize and do what they feel able doing as soon as they can, but that heavy lifting and strenuous exercise should be avoided for 4 weeks after surgery^35^.

### Surgical-site occurrences

The term surgical-site occurrences was introduced by the Ventral Hernia Working Group and includes not only surgical-site infections but also seromas, haematomas, wound dehiscence, and enterocutaneous fistulas^12,126^. MIS repairs have a lower risk of surgical-site occurrences than open procedures^35^. Most surgical-site occurrences are mild and will resolve by themselves within weeks to months. However, it seems that early wound infection is associated with an increased risk of recurrence in the long term^4,127^.

A deep surgical-site infection, including the mesh, is a severe and difficult complication of ventral hernia repair^128^. Macroporous monofilament polypropylene meshes are associated with low infection rates and increased mesh salvage rates^129^. When a mesh infection occurs, mesh salvage may be successful by using long-course antibiotics, percutaneous drainage, wound debridement, and negative pressure dressings. When attempting to salvage the mesh, consider the degree of mesh incorporation, mesh exposure, bowel integrity, and the patient’s clinical condition. When the mesh cannot be salvaged, mesh explantation can become necessary^128^.

### Persistent pain

Postoperative pain usually resolves within 3 months after surgery. Pain may be caused by seroma or haematoma formation, sutures or tacks, mesh infection, recurrence, nerve damage, or by the surgical trauma itself. Chronic pain is defined as pain that persists longer than 6 months and is reported to be around 5–15% after umbilical and incisional hernia repair^130–132^. CT imaging may be needed for evaluation. When no definite cause is seen using CT imaging, several strategies, including analgesics, anti-inflammatory medications, steroids, and nerve blocks, can be tried. Lastly, removal of tacks or transfascial sutures in the area of pain and, in some cases, removal of the mesh may be necessary.

### Recurrence

Hernia recurrence is an often used measure of evaluating the performance of a particular surgical technique for ventral hernia repair. It is well known that repair without mesh, as well as postoperative surgical-site occurrences, are risk factors associated with increased recurrence rates^4,35,127^. Recurrence rates after ventral hernia repair are reported to be around 15%, depending on follow-up time and evaluation of possible recurrence^130,133^.

## Conclusion

Surgeons and patients should understand that the main aim of treating ventral hernias is to improve the quality of life of patients. The risks and benefits of procedures should be weighed against patients’ complaints and co-morbidities. Addressing and optimizing patient-related risk factors before surgery is important, with a strong emphasis on weight loss, smoking cessation, and diabetic control. Surgeons should be aware of their own limitations and those of their institution, referring more complex cases to specialized surgeons or centres as needed.

## Data Availability

Not applicable.
